# Two new eriophyid mite species associated with Clematis terniflora var. mandshurica in China (Acari, Eriophyidae)

**DOI:** 10.3897/zookeys.621.9443

**Published:** 2016-10-03

**Authors:** Yan Dong, Yan-Mei Sun, Xiao-Feng Xue

**Affiliations:** 1 Department of Entomology, Nanjing Agricultural University, Nanjing, Jiangsu 210095, China; 2 Department of Plant Protection, Jilin Agricultural Science and Technology University, Jilin, Jilin 132101, China

**Keywords:** Aculops, Phyllocoptes, plant feeding, taxonomy

## Abstract

Two new eriophyid mite species associated with Clematis terniflora var. mandshurica, namely *Aculops jilinensis* **sp. n.** and *Phyllocoptes terniflores* **sp. n.**, are described. Both species infest the tender leaves of host plants, inducing severe curling and blistering.

## Introduction

Clematis terniflora DC. var. mandshurica (Rupr.) Ohwi, called “la liao tie xian lian” in Mandarin, belongs to the family Ranunculaceae, and is native to China (Heilongjiang, Jilin, Liaoning, Shanxi, Inner Mongolia), Korea, Mongolia, and the Russian Far East (website of Flora Republicae Popularis Sinicae – http://frps.eflora.cn/frps/Clematis%20terniflora and Germplasm Resources Information Network – https://npgsweb.ars-grin.gov/gringlobal/taxonomydetail.aspx?id=404246). It is also known in folk medicine and planted as an ornamental plant in the northeast of China.

To date, no eriophyid mite species were reported from Clematis terniflora var. mandshurica, although at least nine species are known from other *Clematis* spp. worldwide, namely *Aceria vitalbae* (Canestrini, 1892) (from *Clematis vitalba* L.), *Calepitrimerus clematisis* Song, Xue & Hong, 2008 (from *Clematis* sp.), *Cupacarus subnotatus* (Nalepa, 1924) (from *Clematis recta* L.), *Epitrimerus flammulae* Gerber, 1901 (from *Clematis flammula* L.), *Phyllocoptes atragenes* Liro, 1941 [from *Clematis alpina* (L.) Mill.], *Phyllocoptes heterogaster* (Nalepa, 1890) (from *Clematis recta*) (Nalepa, 1891), *Phyllocoptes heteronotus* Nalepa, 1924 (from *Clematis recta*), *Phyllocoptes monochetus* Nalepa, 1924 (from *Clematis recta*), and *Platyphytoptus vitalbae* Farkas, 1960 (from *Clematis vitalba*).

**Figure 1.**
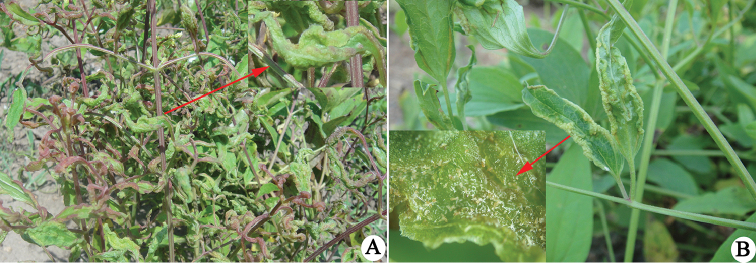
Damage symptoms associated with *Aculops jilinensis* sp. n. and *Phyllocoptes terniflores* sp. n. on Clematis terniflora var. mandshurica: **A** leaves have severely curled and blistered, red arrow indicates the magnified curled leaf **B** leaves have moderately curled and blistered, red arrow indicates part of the mite population hidden inside the curled surface.

During field surveys in 2015, some leaves were found to be severely curled and blistered (Figure 1). The curled and blistered leaves were checked with the aid of a microscope in the laboratory. Eriophyid mites were found and two new species were identified by the first and third authors.

## Materials and methods

Sampling was made on the host plants in the field by the aid of a hand lens (30×), in Jilin Agricultural Science and Technology University, Jilin City, Jilin Province, China. The eriophyid mites gathered with host plant were placed in vials and stored in 75% ethanol, each vial was marked with the collection data. In the laboratory, the samples including mites were poured into a Petri dish and mite specimens were picked up using a fine pin and placed in Keifer’s Booster to clear them. They were then slide-mounted with modified Berlese medium (Amrine and Manson 1996).

Specimens were examined with the aid of a Zeiss A2 (Germany) research microscope with phase contrast (A-plan phase contrast objectives: ×10/0.25 (NA), ×20/0.45 (NA); EC plan-NEOFLUAR phase objectives: ×40/0.75 (NA); ×100/1.3 (NA), oil immersion) and schematic drawings were made. Micrographs were taken at ×100 magnification (×10 eyepiece magnification) with a camera AxioCam MRc attached to the microscope, and connected to a computer, using Axiovision (4.8.2) image analysis software.

The morphological terminology used follows Lindquist (1996) and the generic classification is made according to Amrine et al. (2003). Specimens were measured following de Lillo et al. (2010). For each species, the holotype female measurement precedes the corresponding range for holotype and paratypes (given in parentheses). All measurements are in micrometres (µm) and represent lengths, when not otherwise specified. All type specimens are deposited as slide-mounted specimens in the Arthropod/Mite Collection of the Department of Entomology, Nanjing Agricultural University (NJAU), Jiangsu Province, China.

## Taxonomy

### Family Eriophyidae Nalepa, 1898 Subfamily Phyllocoptinae Nalepa, 1892

#### Tribe Anthocoptini Amrine and Stasny, 1994 Genus *Aculops* Keifer, 1966b

##### Aculops jilinensis sp. n.

Taxon classificationAnimaliaProstigmataEriophyidae

http://zoobank.org/A3F6DB8B-8143-4DFC-990E-CD2EAB8BC505

Figs 2 , 3

###### Diagnosis.

Body fusiform; prodorsal shield with acuminate frontal lobe, median and admedian lines complete and connected at base by a pair of short lines, forming an “arrow”, submedian lines connected by a pair of diagonally reaching lines; scapular seta short 15 (14–20) on rear shield margin, projecting posteriorly; opisthosoma dorsally with evenly curved annuli (54–73 dorsally, 71–84 ventrally) and all standard setae for the Eriophyidae; legs with standard setae, empodium simple, 7-rayed; coxigenital region with three pairs of setae and many granules, female genital coverflap with 13 (12–13) longitudinal ridges and two to three transverse lines at base.

###### Description.

**Figure 2.**
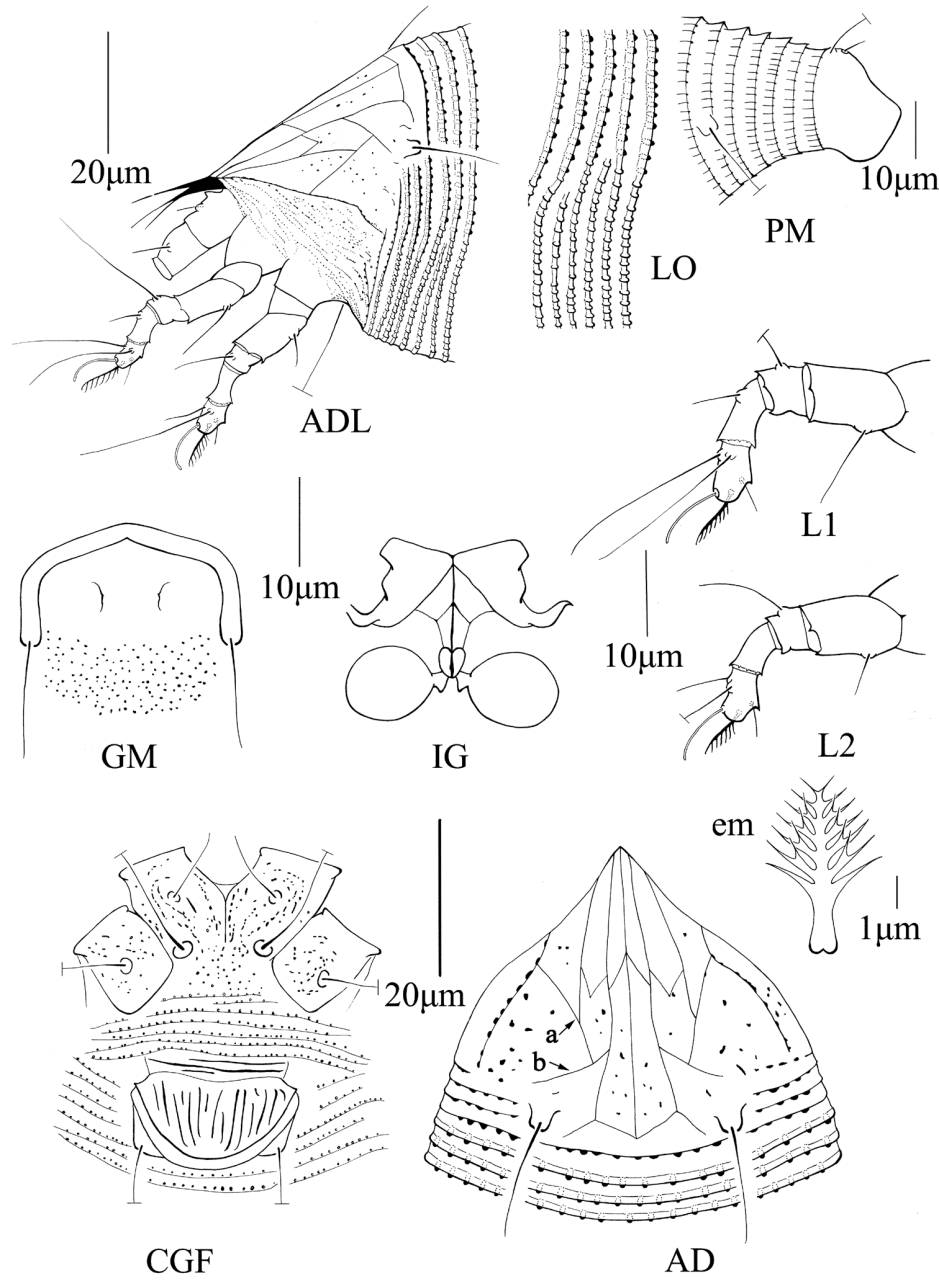
Schematic drawings of *Aculops jilinensis* sp. n.: **ADL** lateral view of anterior body region (slightly rotated dorsad) **LO** lateral view of annuli at mid-region of opisthosoma **PM** lateral view of posteriormost region of opisthosoma **GM** male genital region **IG** female internal genitalia **L1** leg I **L2** leg II **em** empodium **CGF** female coxigenital region **AD** prodorsal shield.

FEMALE: (n = 9). Body fusiform, opisthosoma broadest 12 annuli posterior of the prodorsal shield, then tapering regularly until its posterior apex; 179 (179–271), 50 (50–70) wide, 53 (50–60) thick; light yellow. ***Gnathosoma*** 19 (19–25), projecting obliquely downwards, pedipalp coxal seta (*ep*) 4 (3–4), dorsal pedipalp genual seta (*d*) simple, 6 (6–9), cheliceral stylets 11 (11–20). ***Prodorsal shield*** 38 (30–42), 45 (35–45) wide, subtriangular; frontal lobe acuminate, 6 (5–7). Median and admedian lines complete and connected at base by a pair of short, almost transversal (slightly oblique) lines, forming an “arrow”; median and admedian lines are also connected at centre by a pair of short, diagonally directed lines, forming an inverted “V”; submedian lines formed by two pairs of incomplete lines (submedian lines I and submedian lines II); submedian lines I reaching about midway, merged with a pair of lines converging posteriorly (‘a’ in Figure 2AD), themselves joining perpendicularly another pair of lines oriented anteromesally (‘b’ in Figure 2AD); submedian lines II flanking lateral edges of shield, joining with lines ‘a’; submedian lines II, together with lines ‘a’ and ‘b’, forming a triangular cell, opened posterolaterally; submedian lines I connected with admedian lines at center by a pair of “V” shaped lines; many granules distributed in the ‘triangular’ cell and between median, admedian and submedian lines. Scapular tubercles on rear shield margin, 24 (23–26) apart, scapular seta (*sc*) 15 (14–20), projecting posterior. ***Coxigenital region*** with 7 (5–10) microtuberculated annuli. Coxal plates with granules throughout; anterolateral seta on coxisternal plate I (*1b*) 9 (7–10), 13 (13–14) apart, proximal seta on coxisternal plate I (*1a*) 43 (35–45), 9 (8–11) apart, proximal seta on coxisternal plate II (*2a*) 48 (40–50), 25 (23–25) apart. Prosternal apodeme 6 (6–8). ***Leg I*** 37 (29–37), femur 9 (9–12), basiventral femoral seta (*bv*) 10 (9–11); genu 7 (5–7), antaxial genual seta (*l*’’) 21 (20–25); tibia 9 (7–9), paraxial tibial seta (*l*’) 6 (6–8), located at 1/3 from dorsal base; tarsus 6 (6–7), paraxial, fastigial, tarsal seta (*ft*’) 19 (18–20), antaxial, fastigial, tarsal seta (*ft*’’) 22 (22–26), paraxial, unguinal, tarsal seta (*u*’) 4 (4–5); empodium (*em*) 9 (7–9), simple, 7-rayed, tarsal solenidion (*ω*) 9 (8–10), rod-like. ***Leg II*** 26 (23–26), femur 9 (9–13), basiventral femoral seta (*bv*) 9 (8–12); genu 4 (4–5), antaxial genual seta (*l*’’) 10 (8–11); tibia 7 (6–8); tarsus 6 (5–6), paraxial, fastigial, tarsal seta (*ft*’) 7 (6–9), antaxial, fastigial, tarsal seta (*ft*’’) 25 (21–25), paraxial, unguinal, tarsal seta (*u*’) 5 (4–5); empodium (*em*) 7 (7–8), simple, 7-rayed, tarsal solenidion (*ω*) 9 (9–10), rod-like. ***Opisthosoma*** dorsally arched, with 56 (54–73) dorsal semiannuli bearing rounded microtubercles except last 7-9th semiannuli with elongated microtubercles; ventrally with 72 (71–84) semiannuli, with (longitudinally) elongated microtubercles. Seta *c*2 30 (30–35) on ventral semiannulus 12 (11–13), 45 (43–50) apart; seta *d* 73 (60–73) on ventral semiannulus 24 (20–28), 33 (30–45) apart; seta *e* 17 (15–20) on ventral semiannulus 42 (40–50), 17 (17–25) apart; seta *f* 25 (22–26) on 6^th^ ventral semiannulus from rear, 18 (16–18) apart. Seta *h1* 5 (4–5), seta *h*2 70 (60–72). ***Female genitalia*** 14 (14–17), 21 (21–24) wide, coverflap with 13 (12–13) longitudinal ridges and two to three transverse lines at base, seta 3*a* 34 (34–45), 16 (16–20) apart.

MALE: (n = 1, dorsal view). Body fusiform, 270, 50 wide; light yellow. ***Gnathosoma*** 25, projecting obliquely downwards, pedipalp coxal seta (*ep*) 3, dorsal pedipalp genual seta (*d*) simple, 5, cheliceral stylets 20. ***Prodorsal shield*** 32, 45 wide, subtriangular, frontal lobe acuminate, 5; shield design similar to that of female. Scapular tubercles on rear shield margin, 26 apart, scapular seta (*sc*) 15, projecting posteriorly. ***Coxigenital region*** with 7 microtuberculated annuli. Coxal plates with granules, anterolateral seta on coxisternal plate I (1*b*) 10, 14 apart, proximal seta on coxisternal plate I (1*a*) 43, 10 apart, proximal seta on coxisternal plate II (2*a*) 40, 27 apart. Prosternal apodeme 7. ***Leg I*** 35, femur 12, basiventral femoral seta (*bv*) 10; genu 7, antaxial genual seta (*l*’’) 23; tibia 9, paraxial tibial seta (*l*’) 7, located at 1/3 from dorsal base; tarsus 7, paraxial, fastigial, tarsal seta (*ft*’) 28, antaxial, fastigial, tarsal seta (*ft*’’) 23, paraxial, unguinal, tarsal seta (*u*’) 5; empodium (*em*) 9, simple, 7-rayed, tarsal solenidion (*ω*) 10, rod-like. ***Leg II*** 30, femur 8, basiventral femoral seta (*bv*) 12; genu 5, antaxial genual seta (*l*’’) 13; tibia 7; tarsus 6, paraxial, fastigial, tarsal seta (*ft*’) 9, antaxial, fastigial, tarsal seta (*ft*’’) 23, paraxial, unguinal, tarsal seta (*u*’) 5; empodium (*em*) 7, simple, 7-rayed, tarsal solenidion (*ω*) 10, rod-like. ***Opisthosoma*** dorsally arched, with 70 semiannuli, with rounded microtubercles on the posterior margin, last 7-9th semiannuli with elongated microtubercles, ventrally with 80 semiannuli, with elongated microtubercles. Seta *c2* 38 on ventral semiannulus 16, 55 apart; seta *d* 65 on ventral semiannulus 28, 45 apart; seta *e* 16 on ventral semiannulus 46, 26 apart; seta *f* 29 on 6^th^ ventral semiannulus from rear, 24 apart. Seta *h1* 5, seta *h2* 55. ***Male genitalia*** 24 wide, seta *3a* 40, 20 apart.

###### Type material.

**Holotype** female (slide number NJAUAcariEriJ8A.1; marked Holotype), from Clematis terniflora var. mandshurica (Ranunculaceae), Jilin Agricultural Science and Technology University, Jilin City, Jilin Province, China, 43°57'16"N, 126°28'58"E, elevation 221m, 19 July 2015, coll. Yan-Mei Sun. **Paratypes** 8 females and 1 male on 9 microscope slides (slide number NJAUAcariEriJ8A.2–8A.10), same collection data and repository as holotype.

###### Relationship to host.

Infesting the tender upper leaves; making leaves severely curled and blistered (Figure 1A); hiding inside the curled surfaces (Figure 1B).

###### Etymology.

The specific designation *jilinensis* is derived from the name of location, Jilin City, where the new species was collected.

###### Remarks.

Up to now, no eriophyoid mite species in the genus *Aculops* was reported from the host plant family Ranunculaceae. The new species is similar to the other species in the genus *Aculops* but can be easily distinguished by characters of specific prodorsal shield design. However, it is mostly similar to *Aculops alachuae* Keifer, 1966b, which also has dorsal annuli with rounded microtubercles, coxal plates with granules, female genital coverflap with longitudinal ridges and prodorsal shield with lined design and many granules. The new species can be separated from *Aculops alachuae* by its 7-rayed empodium (4-rayed in *Aculops alachuae*), median and admedian lines connected at the base by a pair of transverse lines forming an “arrow” (an “arrow” is present at the base of median line, but not connected with admedian lines in *Aculops alachuae*), submedian lines connected by diagonally reaching lines (submedian lines separated in *Aculops alachuae*), scapular seta *sc* short, 15 (14–20) (seta *sc* 27 in *Aculops alachuae*). *Aculops alachuae* was reported infesting Rhus copallinum L. var. leucantha (Jacq.) DC. (Anacardiaceae) from Florida, USA, galling host plant leaves (Keifer, 1966b). The new species is also similar to *Aculops euphorbicolus* (Keifer, 1964), which also have annuli with rounded microtubercles (53 dorsal annuli), coxal plates with granules, female genital coverflap with longitudinal ridges and prodorsal shield with lined design, 7-rayed empodium, but can be differentiated by prodorsal shield with many granules between lines (prodorsal shield without granules in *Aculops euphorbicolus*), median line complete (median line incomplete in *Aculops euphorbicolus*), opisthosoma with 72 (71–84) ventral annuli (opisthosoma with 60 ventral annuli in *Aculops euphorbicolus*), female genital coverflap with 13 (12–13) longitudinal ridges and two to three transverse lines at base (female genital coverflap with 8–10 ridges and two rows of granules in *Aculops euphorbicolus*). *Aculops euphorbicolus* was reported from *Euphorbia corollata* L. (Euphorbiaceae) from Virginia (USA), making deformed flower clusters or galls (Keifer, 1964).

**Figure 3.**
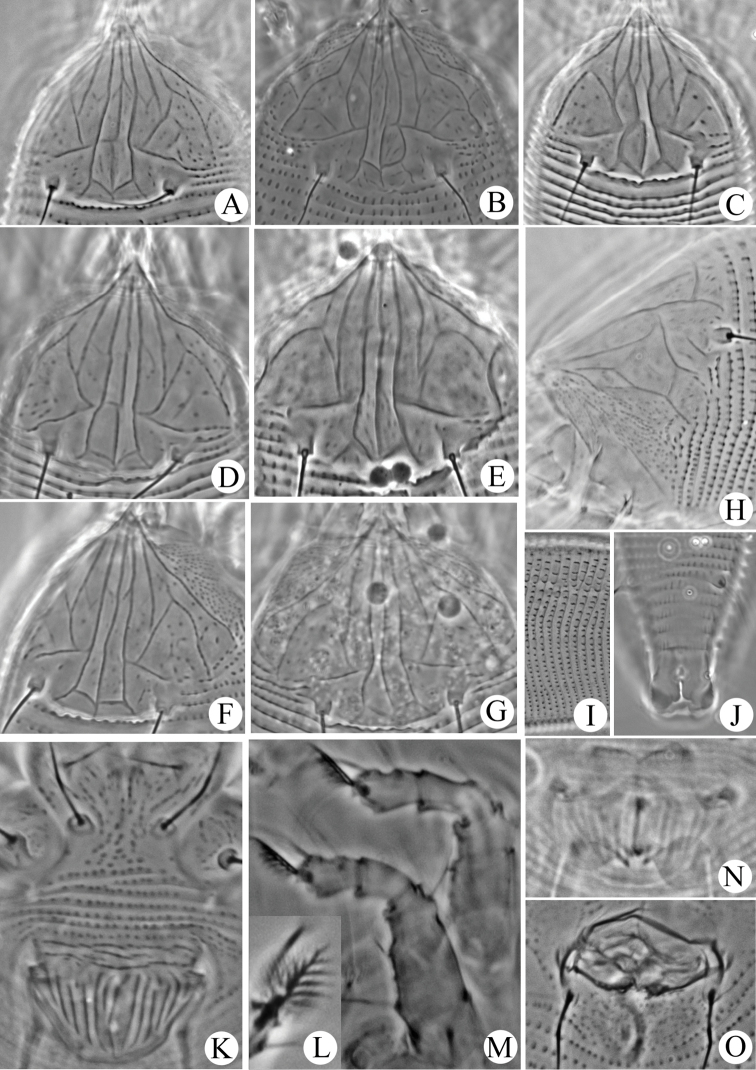
Micrographs of *Aculops jilinensis* sp. n.: **A–G** prodorsal shield **H** lateral view of anterior body region **I** lateral view of annuli at mid-region of opisthosoma **J** ventral view of posteriormost region of opisthosoma **K** female coxigenital region **L** empodium **M** legs I (right) and II (left) **N** female internal genitalia **O** male genital region.

Some intraspecific differences in the design of the prodorsal shield were observed, especially between the median and admedian lines. The median line is complete in all specimens examined except the specimen illustrated in Figure 3C (median line interrupted at centre). Besides connected at base, median and admedian lines are always separated in specimens in Figures 3E and G. Median and admedian lines connected at base, basal 2/3 and 1/3 in Figures 3A, B, D, and F.

#### Tribe Phyllocoptini Nalepa, 1892 Genus *Phyllocoptes* Nalepa, 1887

##### Phyllocoptes terniflores sp. n.

Taxon classificationAnimaliaProstigmataEriophyidae

http://zoobank.org/BA0B864A-96A5-48C4-A9BC-C2F3F499364E

Figs 4 , 5

###### Diagnosis.

Body fusiform; prodorsal shield with broad frontal lobe, scapular setae ahead of rear shield margin, projecting upward-centrally, median, admedian and submedian lines formed by granules aligned and making a network; opisthosoma dorsally with three ridges, middorsal ridge fade as long as lateral ridges, with 55 (55–60) dorsal and 100 (92–100) ventral annuli, all standard setae of the Eriophyidae present; legs with standard setae, empodium simple, 5-rayed; coxigenital region with three pairs of setae and many granules, female genital coverflap with 12 (10–12) longitudinal ridges and two transverse lines at the base.

###### Description.

FEMALE: (n = 10). Body fusiform, 256 (200–304), 73 (68–78) wide, opisthosoma broadest 12–13 annuli posterior of the prodorsal shield, then tapering regularly until its posterior apex; light yellow. ***Gnathosoma*** 25 (20–26), projecting obliquely downwards, pedipalp coxal seta (*ep*) 3 (3–4), dorsal pedipalp genual seta (*d*) simple, 9 (7–9), cheliceral stylets 15 (15-22). ***Prodorsal shield*** 50 (50–55), 57 (55–65) wide, semicircular; frontal lobe broad, 7 (6–7). Shield pattern composed of granules aligned and connected by faint lines. Median line: largely broken at centre; anterior part originated on the frontal lobe and ended at about 1/5 of the anterior prodorsal shield, thereafter, connected with admedian lines by a pair of transverse lines; posterior part originated at about 4/5 of prodorsal shield, vanished at rear of prodorsal shield, connected with admedian lines by a pair of transverse line at anterior. Admedian lines complete and sinuous and connected with submedian lines by two pairs of transverse lines at basal 3/4 and center of prodorsal shield. Submedian lines flanking lateral edges of shield branched into two curled lines, forming a large open semicircle at lateral side of prodorsal shield; many aligned granules distributed between submedian lines. Scapular tubercles ahead of rear shield margin, 22 (22–26) apart, scapular seta (*sc*) 12 (12–14), projecting upward-centrally. ***Coxigenital region*** with 13 (11–13) microtuberculated annuli. Coxal plates with granules and irregular lines throughout, anterolateral seta on coxisternal plate I (*1b*) 15 (13–15), 15 (15–17) apart, proximal seta on coxisternal plate I (*1a*) 45 (35–45), 12 (11–14) apart, proximal seta on coxisternal plate II (*2a*) 65 (63–65), 32 (31–35) apart. Prosternal apodeme 6 (6–7). ***Leg I*** 36 (36–38), femur 13 (13–15), basiventral femoral seta (*bv*) 13 (13–14); genu 7 (6–8), antaxial genual seta (*l*’’) 38 (35–40); tibia 12 (10–12), paraxial tibial seta (*l*’) 7 (5–7), located at 1/3 from dorsal base; tarsus 7 (7–8), paraxial, fastigial, tarsal seta (*ft*’) 17 (17–20), antaxial, fastigial, tarsal seta (*ft*’’) 25 (25–28), paraxial, unguinal, tarsal seta (*u*’) 5 (4–5); empodium (*em*) 8 (7–8), simple, 5-rayed, tarsal solenidion (*ω*) 8 (7–8), knobbed. ***Leg II*** 35 (33–35), femur 14 (13–15), basiventral femoral seta (*bv*) 15 (12–15); genu 6 (5–6), antaxial genual seta (*l*’’) 9 (6–9); tibia 10 (8–10); tarsus 6 (6–7), paraxial, fastigial, tarsal seta (*ft*’) 5 (5–6), antaxial, fastigial, tarsal seta (*ft*’’) 24 (24–28), paraxial, unguinal, tarsal seta (*u*’) 5 (4–5); empodium (*em*) 8 (7–8), simple, 5-rayed, tarsal solenidion (*ω*) 9 (9–10), knobbed. ***Opisthosoma*** dorsally with 55 (55–60) semiannuli, with rounded microtubercles on the posterior margin, except last 6th semiannuli with elongated microtubercles; ventrally with 100 (92–100) semiannuli, with nearly rounded microtubercles on central part; moreover, with elongated microtubercles in side area and the ventral semiannulus between seta *e* and *f*; last 6 ventral semiannuli with elongated and linear microtubercles. Seta *c2* 40 (40–43) on ventral semiannulus 21 (20–22), 67 (65–74) apart; seta *d* 54 (43–45) on ventral semiannulus 43 (39–43), 48 (47–50) apart; seta *e* 32 (29–32) on ventral semiannulus 69 (63–69), 26 (24–26) apart; seta *f* 37 (36–37) on 6^th^ ventral semiannulus from rear, 28 (25–28) apart. Seta *h1* 5 (4–5), seta *h2* 87 (83–87). ***Female genitalia*** 17 (17–20), 24 (24–27) wide, coverflap with 12 (10–12) longitudinal ridges and two transverse lines at the base, seta 3*a* 27 (23–28), 19 (19–21) apart.

MALE: (n = 1). Body fusiform, 233, 72 wide; white. ***Gnathosoma*** 20, projecting obliquely downwards, pedipalp coxal seta (*ep*) 3, dorsal pedipalp genual seta (*d*) simple, 7, cheliceral stylets 15. ***Prodorsal shield*** 50, included the frontal lobe, 55 wide, with a broad based frontal lobe broad, 8, shield design similar to that of female. Scapular tubercles ahead of rear shield margin, 22 apart, scapular seta (*sc*) 11, projecting centrad. ***Coxigenital region*** with 14 microtuberculated annuli. Coxal plates with irregular lines, anterolateral seta on coxisternal plate I (*1b*) 9, 14 apart, proximal seta on coxisternal plate I (*1a*) 33, 10 apart, proximal seta on coxisternal plate II (2*a*) 50, 28 apart. Prosternal apodeme 10. ***Leg I*** 34, femur 12, basiventral femoral seta (*bv*) 9; genu 5, antaxial genual seta (*l*’’) 27; tibia 10, paraxial tibial seta (*l*’) 6, located at 1/3 from dorsal base; tarsus 7, paraxial, fastigial, tarsal seta (*ft*’) 16, antaxial, fastigial, tarsal seta (*ft*’’) 24, paraxial, unguinal, tarsal seta (*u*’) 4; empodium (*em*) 7, simple, 5-rayed, tarsal solenidion (*ω*) 8, knobbed. ***Leg II*** 27, femur 11, basiventral femoral seta (*bv*) 10; genu 10, antaxial genual seta (*l*’’) 6; tibia 9; tarsus 6, paraxial, fastigial, tarsal seta (*ft*’) 5, antaxial, fastigial, tarsal seta (*ft*’’) 24, paraxial, unguinal, tarsal seta (*u*’) 5; empodium (*em*) 6, simple, 5-rayed, tarsal solenidion (*ω*) 8, knobbed. ***Opisthosoma*** dorsally with 45 semiannuli, with rounded microtubercles on the posterior margin, last 5^th^ semiannuli with elongated microtubercles; ventrally with 82 semiannuli, with nearly rounded microtubercles on central part; moreover, with elongated microtubercles in side area and the ventral semiannulus between seta *e* and *f*; last six ventral semiannuli with elongated and linear microtubercles. Seta *c2* 30 on ventral semiannulus 18, 62 apart; seta *d* 30 on ventral semiannulus 30, 40 apart; seta *e* 20 on ventral semiannulus 50, 23 apart; seta *f* 26 on 6^th^ ventral semiannulus from rear, 23 apart. Seta *h1* 5, seta *h2* 60. ***Male genitalia*** 19 wide, seta *3a* 14.

**Figure 4.**
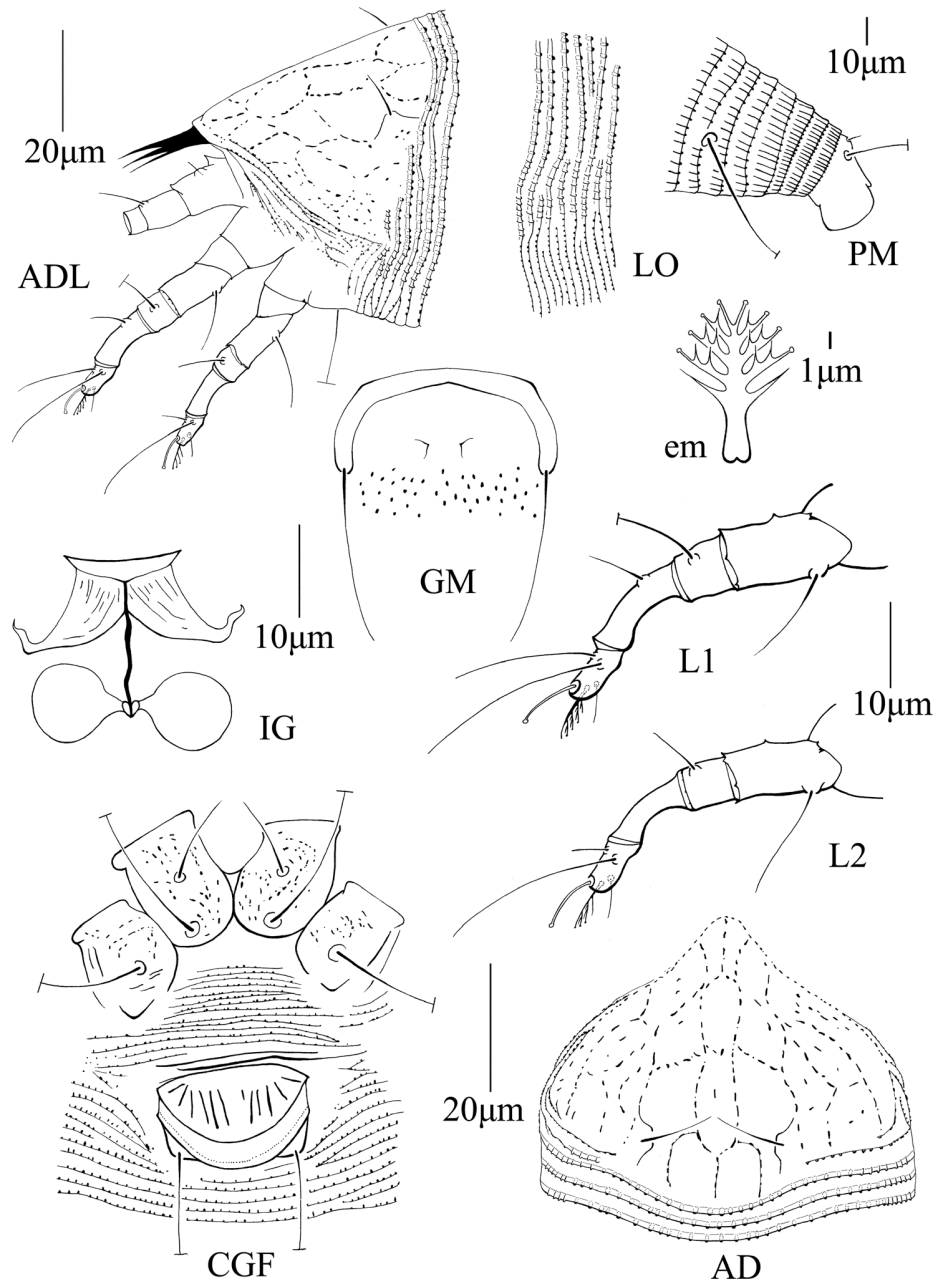
Schematic drawings of *Phyllocoptes terniflores* sp. n.: **ADL** lateral view of anterior body region (slightly rotated dorsad) **LO** lateral view annuli **PM** lateral view of posterior opisthosoma **em** empodium **GM** male genital region **IG** female internal genitalia **L1** leg I **L2** leg II **CGF** female coxigenital region **AD** prodorsal shield.

###### Type material.

**Holotype** female (slide number NJAUAcariEriJ8B.1; marked Holotype), from Clematis terniflora var. mandshurica (Ranunculaceae), Jilin Agricultural Science and Technology University, Jilin City, Jilin Province, China, 43°57'16"N, 126°28'58"E, elevation 221m, 19 July 2015, coll. Yan-Mei Sun. **Paratypes** 9 females and 1 male on ten microscope slides (slide number NJAUAcariEriJ8B.2–8B.11), same collection data and repository as holotype.

###### Relationship to host.

Infesting the tender upper leaves and making leaves severely curled and blistered (Figure 1A); hiding inside the curled surfaces (Figure 1B).

###### Etymology.

The specific designation *terniflores* is derived from the species name of the host plant, *terniflora*.

###### Remarks.

The new species was compared with others in the genus *Phyllocoptes* infesting *Clematis* sp. This species is similar to *Phyllocoptes atragenes* [from *Clematis alpina*, infesting host plant as curled leaves], but can be differentiated from the latter by its shield pattern: the median and admedian lines are discontinuous (median and admedian lines continuous in *Phyllocoptes atragenes*), dorsal opisthosoma with 55 (55–60) annuli (dorsal opisthosoma with 48 annuli in *Phyllocoptes atragenes*) and dorsal annuli with rounded microtubercles (dorsal annuli smooth in *Phyllocoptes atragenes*). This species is also similar to *Phyllocoptes heterogaster* (Nalepa, 1891) [from *Clematis recta*, infesting host plant as abnormal hair], but can be differentiated from the latter by having its coxal plates with granules and short lines (coxal plates smooth in *Phyllocoptes heterogaster*), empodium 5-rayed (empodium 4-rayed in *Phyllocoptes heterogaster*), median line present on anterior of prodorsal shield (median line absent from anterior of prodorsal shield in *Phyllocoptes heterogaster*).

Besides species from *Clematis* sp., the new species is also similar to *Phyllocoptes calirubi* Keifer, 1938 [from *Rubus ursinus* Cham. & Schltdl. (Rosaceae)], *Phyllocoptes exochordae* Keifer, 1972 [from *Exochorda racemosa* (Lindl.) Rehder (Rosaceae)] and *Phyllocoptes neenachensis* Keifer, 1966a [from *Oenothera deltoides* Torr. & Frém. (Onagraceae)] by dorsal and ventral annuli with rounded microtubercles, female genital coverflap with longitudinal ridges and especially prodorsal shield design formed by granules aligned (besides with the generic characters of *Phyllocoptes*). The new species can be differentiated from the later three species by large size of body, 256 (200–304) (140–155 in *Phyllocoptes calirubi*, 200–215 in *Phyllocoptes exochordae* and 145–195 in *Phyllocoptes neenachensis*), median line present at anterior of dorsal shield (median lines absent from anterior of dorsal shield in all three species), coxal area with many granules and short lines (coxal area with few short lines in *Phyllocoptes calirubi*; coxal area I with short lines, coxal area II smooth in *Phyllocoptes exochordae*; coxal area with granules and short lines in *Phyllocoptes neenachensis*), solenidion knobbed (solenidion unknobbed in all three species), empodium 5-rayed (empodium 5-rayed in *Phyllocoptes calirubi*, empodium 6-rayed in *Phyllocoptes exochordae* and empodium 4-rayed in *Phyllocoptes neenachensis*) and short scapular seta 7 (6–7) (scapular seta 11 in *Phyllocoptes calirubi* and *Phyllocoptes exochordae*, scapular seta 10 in *Phyllocoptes neenachensis*).

**Figure 5.**
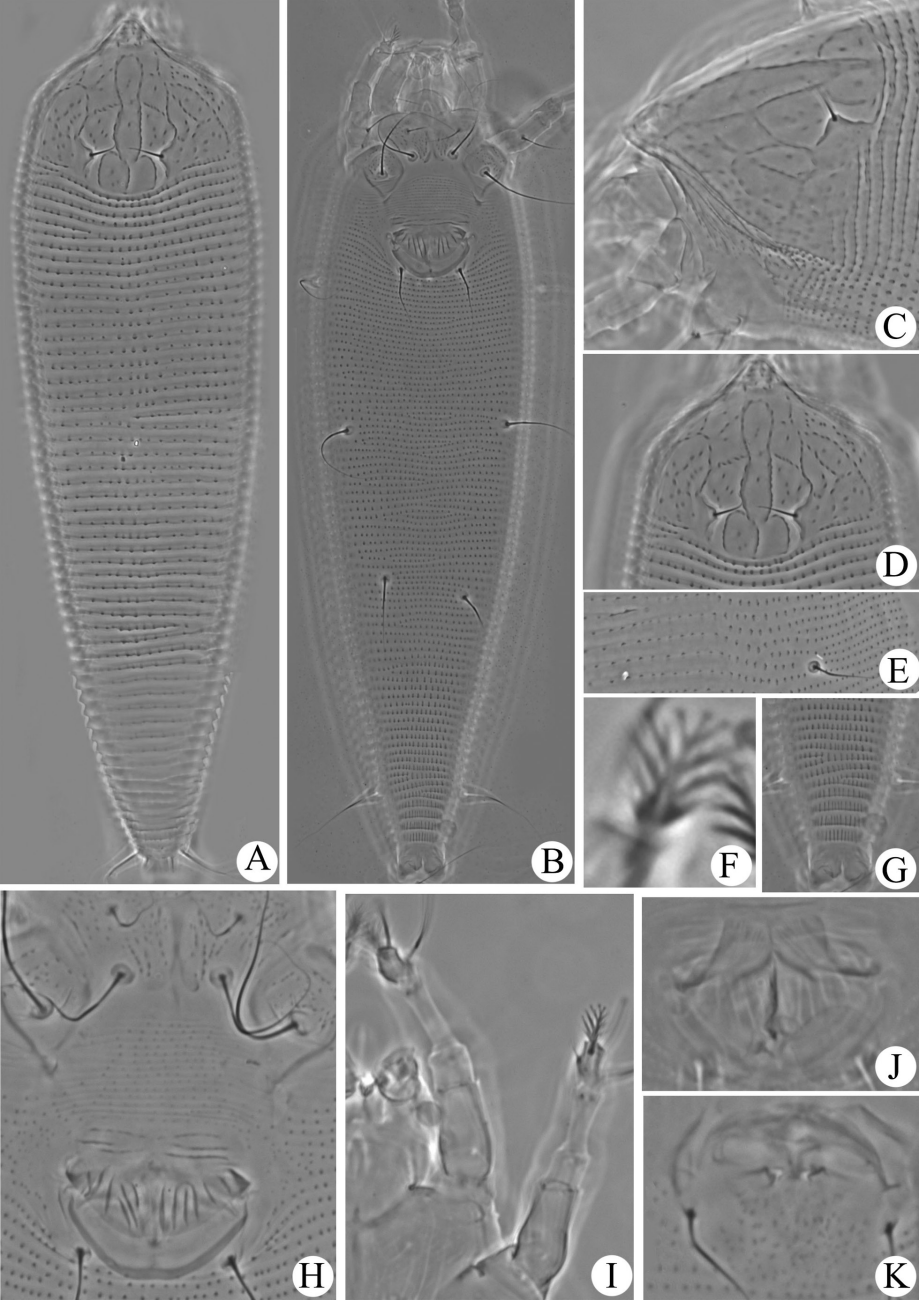
Micrographs of *Phyllocoptes terniflores* sp. n.: **A** dorsal view of female **B** ventral view of female **C** lateral view of anterior body region **D** prodorsal shield **E** lateral view annuli **F** empodium **G** ventral view of posterior opisthosoma **H** coxigenital region and female genitalia **I** leg I and leg II **J** female internal genitalia **K** male genital region.

## Supplementary Material

XML Treatment for Aculops jilinensis

XML Treatment for Phyllocoptes terniflores
